# *Notes from the Field:* Rollout of Nirsevimab to Protect Infants and Young Children During the Respiratory Syncytial Virus Season — New York City, 2023–2024

**DOI:** 10.15585/mmwr.mm7348a4

**Published:** 2024-12-05

**Authors:** Melanie S. Askari, Kristin Oliver, Denise Benkel, Melissa Mickle-Hope, Vincent Tam, Marisa Langdon-Embry, Georgia Elysee, Bindy Crouch

**Affiliations:** ^1^New York City Health Department, New York, New York; ^2^Epidemic Intelligence Service, CDC.

**Summary**

**What is already known about this topic?**

Nirsevimab, a monoclonal antibody that protects infants and young children against severe respiratory syncytial virus (RSV) infection through passive immunization, was approved for U.S. use during the 2023–24 RSV season.

**What is added by this report?**

Among New York City infants who were born during the recommended nirsevimab administration period for the 2023–24 RSV season (October 1, 2023–March 31, 2024) and who reportedly received nirsevimab, 37% of Vaccines for Children (VFC)–eligible and 45% of non–VFC-eligible infants received it within the first 7 days of life.

**What are the implications for public health practice?**

Ensuring birthing hospital VFC enrollment and establishing protocols to offer nirsevimab to eligible infants before hospital discharge might increase nirsevimab administration within the first week of life.

Respiratory syncytial virus (RSV) infection is the leading cause of infant hospitalizations in the United States (*1*). During the 2023–24 RSV season, nirsevimab, a new, long-acting injectable human recombinant monoclonal antibody that prevents severe RSV infection in infants and young children became available. In August 2023, the Advisory Committee on Immunization Practices recommended administration of nirsevimab to infants aged 0–7 months and to infants and children aged 8–19 months with risk factors for severe RSV disease, shortly before the RSV season, or within the first week of life if born during October–March in most of the United States (*2*,*3*).

A nationwide shortage of nirsevimab in October 2023 affected the commercial market and the federally funded Vaccines for Children (VFC) program*^,^^†^; in response to the shortage, CDC issued a health advisory recommending prioritization of available nirsevimab doses for infants at highest risk for severe RSV disease, which necessitated a revised VFC allocation strategy (*4*). In New York City (NYC), approximately 75% of children are eligible for public, no-cost immunizations distributed by the NYC Health Department’s VFC program. During the 2023–24 RSV season, approximately two thirds of NYC birthing hospitals^§^ were enrolled in VFC (26 of 38 birthing hospitals).

This analysis examined reported administration of nirsevimab doses, stratified by VFC eligibility status, to determine whether reported doses aligned with NYC’s 2023–24 RSV season nirsevimab VFC distribution strategy. This strategy prioritized reaching VFC-eligible infants during the first week of life at birthing hospitals. 

## Investigation and Outcomes

Nirsevimab doses (15,521) administered during October 1, 2023–March 31, 2024, to infants and children aged 0–19 months, and reported to the NYC Citywide Immunization Registry, which serves as the immunization information system (IIS) for NYC, were characterized. Distribution of doses by patient demographic characteristics, period of administration in relation to the shortage, and facility setting was determined overall and by VFC eligibility status. Among those infants who were born during the recommended nirsevimab administration period for the 2023–24 RSV season (October 1, 2023–March 31, 2024) and who reportedly received nirsevimab, the proportions of infants and children who received nirsevimab within and after the first 7 days of life were determined overall and by VFC eligibility status. VFC eligibility status was determined based on IIS-reported insurance status; infants and children were considered VFC-eligible if they were enrolled in Medicaid, were covered by Child Health Plus B insurance or Section 317 Immunization Program funding, were uninsured or underinsured, or were Native American or Alaskan Native.^¶^ This activity was reviewed by CDC, deemed not research, and was conducted consistent with applicable federal law and CDC policy.**

Overall, 15,521 nirsevimab doses were administered to infants and children aged 0–19 months and reported to IIS in NYC shortly before or during the 2023–24 RSV season (Table). Among the subset of infants who were born during the recommended administration period for the 2023–24 RSV season (October 1, 2023–March 31, 2024) and who received nirsevimab (13,812), 45% received nirsevimab within the first 7 days of life, including 37% of VFC-eligible infants, 45% of non–VFC-eligible infants and children, and 61% of infants whose VFC eligibility was missing or unknown. Among VFC-eligible infants and children aged 0–19 months who received nirsevimab, 18% received nirsevimab at their birthing hospital, compared with 8% of non–VFC-eligible infants and children and 38% of those with missing or unknown VFC eligibility.

**TABLE:** Characteristics of infants and children aged 0–19 months who received nirsevimab immunizations and had their doses reported to the immunization information system, by Vaccines for Children program eligibility status — New York City,*^,^**^†^** 2023–24 respiratory syncytial virus season

Characteristic	No. (column %)			
	Overall N = 15,521	VFC-eligible n = 7,000	Non–VFC-eligible n = 5,678	VFC eligibility missing or unknown^§^ n = 2,843
**Patient age, days**				
Mean (SD)	**60.4 (82.5)**	72.5 (88.4)	58.4 (80.0)	34.7 (64.4)
Median (range)	**23.0 (0–605)**	36.0 (0–603)	21.0 (0–605)	3.0 (0–571)
**Early life nirsevimab administration^¶^ (n = 13,812)**				
Within first 7 days of life	**6,161 (44.6)**	2,231 (36.8)	2,276 (45.0)	1,654 (61.3)
After first 7 days of life	**7,651 (55.4)**	3,825 (63.2)	2,782 (55.0)	1,044 (38.7)
**Age at administration, mos**				
0–7	**15,123 (97.4)**	6,767 (96.7)	5,561 (97.9)	2,795 (98.3)
8–19	**398 (2.6)**	233 (3.3)	117 (2.1)	48 (1.7)
Period of administration**				
Before shortage	**471 (3.0)**	65 (0.9)	356 (6.3)	50 (1.8)
After shortage	**8,540 (55.0)**	3,359 (48.0)	3,621 (63.8)	1,560 (54.9)
Return to routine eligibility	**6,510 (41.9)**	3,576 (51.1)	1,701 (30.0)	1,233 (43.4)
**Race and ethnicity^††^**				
Asian or Pacific Islander	**2,539 (16.4)**	1,268 (18.1)	920 (16.2)	351 (12.3)
Black or African American	**1,964 (12.7)**	1,031 (14.7)	504 (8.9)	429 (15.1)
White	**5,053 (32.6)**	1,167 (16.7)	2,919 (51.4)	967 (34.0)
Hispanic or Latino	**5,048 (32.5)**	3,228 (46.1)	964 (17.0)	856 (30.1)
Other or unknown	**785 (5.1)**	273 (3.9)	307 (5.4)	205 (7.2)
Multiracial	**132 (0.9)**	33 (0.5)	64 (1.1)	35 (1.2)
**Neighborhood poverty level**				
<10% FPL, low poverty	**3,108 (20.0)**	731 (10.4)	1,783 (31.4)	594 (20.9)
≥10% to <20% FPL	**6,334 (40.8)**	2,951 (42.2)	2,333 (41.1)	1,050 (36.9)
≥20% to <30% FPL	**2,767 (17.8)**	1,598 (22.8)	711 (12.5)	458 (16.1)
≥30% to 100% FPL, very high poverty	**2,121 (13.7)**	1,330 (19.0)	325 (5.7)	466 (16.4)
Missing	**1,191 (7.7)**	390 (5.6)	526 (9.3)	275 (9.7)
**Nirsevimab dose**				
50 mg	**11,033 (71.1)**	4,486 (64.1)	4,109 (72.4)	2,438 (85.8)
100 mg	**4,488 (28.9)**	2,514 (35.9)	1,569 (27.6)	405 (14.2)
**Facility setting**				
FQHC or FQHC look-alike^§§^	**1,535 (9.9)**	1,279 (18.3)	149 (2.6)	107 (3.8)
Private ambulatory	**4,860 (31.3)**	1,461 (20.9)	2,647 (46.6)	752 (26.5)
Hospital ambulatory	**2,576 (16.6)**	1,667 (23.8)	690 (12.2)	219 (7.7)
Birthing hospital	**2,820 (18.2)**	1,269 (18.1)	470 (8.3)	1,081 (38.0)
Hospital pediatric ward, nonbirthing hospital	**357 (2.3)**	185 (2.6)	84 (1.5)	88 (3.1)
Hospital, other	**89 (0.6)**	79 (1.1)	8 (0.1)	2 (0.1)
Other nonprofit ambulatory	**86 (0.6)**	71 (1.0)	2 (0)	13 (0.5)
Hospital unknown, potential setting reporting error^¶¶^	**3,188 (20.5)**	979 (14.0)	1,628 (28.7)	581 (20.4)
Outside New York City	**10 (0.1)**	10 (0.1)	0 (—)	0 (—)

**Abbreviations:** FPL = federal poverty level; FQHC = federally qualified health center; RSV = respiratory syncytial virus; VFC = Vaccines for Children program.

* This study is a patient-level analysis. Analyses represent first doses only. In total, 181 nirsevimab second doses were not included.

^†^ A total of 87 nirsevimab doses reported as administered to patients aged >19 months were excluded from this analysis, which was restricted to the age eligibility criteria recommended for nirsevimab by the Advisory Committee on Immunization Practices.

^§^ The substantial proportion of infants immunized in birthing hospitals with missing or unknown VFC eligibility might reflect uncertainty of newborn VFC eligibility status in these settings.

^¶^ Among infants born during the recommended nirsevimab administration period for the 2023–24 RSV season (i.e., October 1, 2023–March 31, 2024).

** Shortage periods were defined based on CDC notifications. Before shortage = October 1–22, 2023; after shortage = October 23, 2023–January 11, 2024; and return to routine eligibility = January 12–March 31, 2024.

^††^ Persons of Hispanic or Latino (Hispanic) origin might be of any race but are categorized as Hispanic; all racial groups are non-Hispanic.

^§§^ An FQHC look-alike meets the FQHC program requirements but does not receive FQHC program funding.

^¶¶^ “Hospital unknown, potential setting reporting error” included adult settings or employee health and setting was likely misreported for these doses.

## Preliminary Conclusions and Actions

NYC IIS data reflect administration of approximately 15,500 nirsevimab doses shortly before or during the 2023–24 RSV season, indicating that many infants and children aged 0–19 months were protected from severe RSV with this highly effective new RSV immunization (*5*). Approximately one half of nearly 14,000 nirsevimab doses reported to the NYC IIS administered to infants born shortly before or during the RSV season were administered within the first week of life. Entering the 2024–25 RSV season, an adequate supply of nirsevimab will be important to continue to protect infants and children. Further, the NYC Health Department continues to prioritize VFC enrollment for the remaining few birthing hospitals not currently enrolled. As of November 2024, a majority of NYC birthing hospitals were enrolled in VFC (31 of 38 birthing hospitals). Ensuring birthing hospital VFC enrollment and establishing protocols to offer nirsevimab to eligible infants before hospital discharge might increase nirsevimab administration within the first week of life.

## Footnotes

* Since 1994, the VFC program, a federal entitlement program, has provided vaccines to eligible uninsured or underinsured persons aged ≤18 years.

† Although nirsevimab is not an active immunizing agent, it is authorized for administration as an immunization under the VFC program.

§ Birthing hospitals are defined as hospitals with labor and delivery units.

¶
https://www.cdc.gov/vaccines-for-children/hcp/program-eligibility/index.html

** 45 C.F.R. part 46, 21 C.F.R. part 56; 42 U.S.C. Sect. 241(d); 5 U.S.C. Sect. 552a; 44 U.S.C. Sect. 3501 et seq.
